# Safety and feasibility of selective tongue fat reduction with injected ice‐slurry

**DOI:** 10.1002/lio2.902

**Published:** 2022-09-09

**Authors:** Michael Ian Orestes, Sara Moradi Tuchayi, Ying Wang, William Farinelli, Knarik Arkun, R. Rox Anderson, Richard Thomas, Lilit Garibyan

**Affiliations:** ^1^ Department of Surgery Uniformed Services University of the Health Sciences Bethesda Maryland USA; ^2^ Wellman Center for Photomedicine, Department of Dermatology Massachusetts General Hospital, Harvard Medical School Boston Massachusetts USA; ^3^ Department of Pathology and Laboratory Medicine Tufts Medical Center Boston Massachusetts USA

**Keywords:** adipose tissue, cryoablation, cryotherapy, obstructive sleep apnea, sleep, upper airway obstruction, tongue fat

## Abstract

**Objectives:**

There is growing evidence that excess adipose tissue within the head and neck contributes to obstructive sleep apnea (OSA), particularly in obese patients. This subset of the population is often difficult to treat with surgical therapies. We theorized that a novel, transcervical method of injectable cryoablation using ice‐slurry can achieve low temperatures without causing neurovascular damage or airway distress in a swine model.

**Methods:**

Four Yorkshire pigs were injected with ice‐slurry comprised of normal saline and 10% glycerol cooled to −6°C via a transcervical, ultrasound guided approach. Direct laryngoscopy was used to confirm accurate placement of the slurry. Thermocouple placement at the needle‐tip was used to measure temperatures at injection site. Swine were monitored for clinical signs of tongue necrosis and airway edema for 2 months, and then euthanized. Twelve biopsy samples from the base of the tongue were collected for histology. These were assessed for presence of tissue damage, inflammation and collagen formation by a blinded board‐certified pathologist.

**Results:**

Tongue tissue temperature below 10°C was achieved for 13.5 ± 1.1 min. Minimum tissue temperature was −4 ± 0.6°C. There was no clinical or pathological evidence of tongue damage to include damage to the lingual nerve or artery. There was some histologic evidence of new collagen formation in areas of the tongue.

**Conclusions:**

Transcervical ultrasound‐guided ice‐slurry injection is feasible, well‐tolerated at temperatures previously shown to be capable of selectively targeting adipose tissue in the base of the tongue in a preclinical swine model, without causing neurovascular damage or airway distress when properly injected.

## Graphical abstract

This is the first animal study investigating the effects of injectable cryoslurry on the tongue. The technology offers a potential minimally invasive, office based procedure for treatment of OSA. This technology may also be useful in the treatment of parapharyngeal and submental fat.

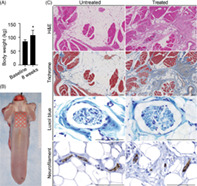

## 1 INTRODUCTION

Obstructive sleep apnea (OSA) is chronic disease which results in daytime sleepiness and cognitive impairment leading to an increased risk of motor vehicle accidents.^1^ The pathophysiology of OSA is complex. Modern concepts of OSA break the pathophysiology into different categories to include: anatomic obstruction resulting in high critical passive closing pressures (Pcrit), inadequate dilation of the dilator muscles during sleep, premature awakening due to an obstruction and an oversensitive ventilatory control system.^2^ When considering surgical options for the treatment of OSA, it is critically important to consider both the patient's phenotype and their anatomy in determining the appropriate surgical therapy.^3^ Patients with high body mass index (BMI), lateral pharyngeal wall collapse and supraglottic laryngeal collapse are likely to suffer from poor surgical outcomes.^3^, ^4^ The reasons why patients with high BMI have OSA are complex and not well understood.^5^ However, recent studies have shown that reduction in tongue adipose tissue correlates with improved disease severity indices following weight loss thus establishing the tongue fat as potential therapeutic target for OSA.^5^, ^6^ Reduction of upper aerodigestive tract fat may improve OSA by increasing the parapharyngeal and retroglossal airway spaces, or by improving protrusion functions of the tongue, offering specific treatment for patients with high Pcrit and would otherwise be difficult to treat surgically. Furthermore, there is some evidence that higher fat content in muscles can reduce their effectiveness.^7^ Targeted therapy to selectively reduce tongue fat may offer a minimally invasive method for treatment in this patient population, compared to other non‐selective methods of tissue destruction such as radiofrequency ablation, cobalation and transoral robotic surgery. The non‐selective nature of currently available tongue ablation treatments limits their use in clinical care.

Controlled topical tissue cooling for selective subcutaneous fat removal is known as cryolipolysis.^8^ This technique relies on the concept that lipid‐rich tissue is preferentially sensitive to cooling, because tissue lipids solidify at temperatures close to 10°C, well above the freezing point of tissue water.^8^ This leads to selective apoptosis and reduction of adipose tissue without damage to surrounding water rich tissue.^8^ Although topical cooling induced cryolipolysis is a safe and effective method of superficial subcutaneous fat removal, it is limited in depth and precision of fat it can target^9^, ^10^ In addition, it takes about 45–60 min of topical cooling to extract enough heat through the skin, to reach temperatures in target tissue required for selective cryolipoysis.^11^

We developed an injectable method of cryolipolysis using biocompatible ice‐slurry to allow precise and efficient targeting of adipose tissue at any depth accessible by needle and showed that it can selectively remove subcutaneous adipose tissue in swine model.^12^ In subcutaneous fat, cryolipolysis could be induced with injection of 30 ml of ice slurry which is able to maintain tissue temperature of less than 10°C for over 10 min (unpublished data). In a recently published first‐in‐human study, ice‐slurry injection was shown to be safe, tolerable, and able to induce cryolipolysis in superficial and deep subcutaneous fat.^10^ Although exact time and temperature dosimetry studies are lacking, published human studies have shown that target tissue temperature below 10°C for 10–15 min are able to induce selective cryolipolysis.^9^, ^11^

In this study, we investigate the safety and feasibility of ice‐slurry injection for selective reduction of adipose tissue in the base of the tongue in large animals. The tongue anatomy of swine and humans is similar, and adipose tissue in the base of the swine tongue makes it an excellent large animal model for this investigation. We hypothesized that ice‐slurry can safely be injected in the base of the swine tongue via ultrasound guided approach resulting in cooling of the target tissue below 10°C for at least 10 min without resulting in airway obstruction, lingual nerve damage or tongue necrosis.

## 2 METHODS

**FIGURE 1:**
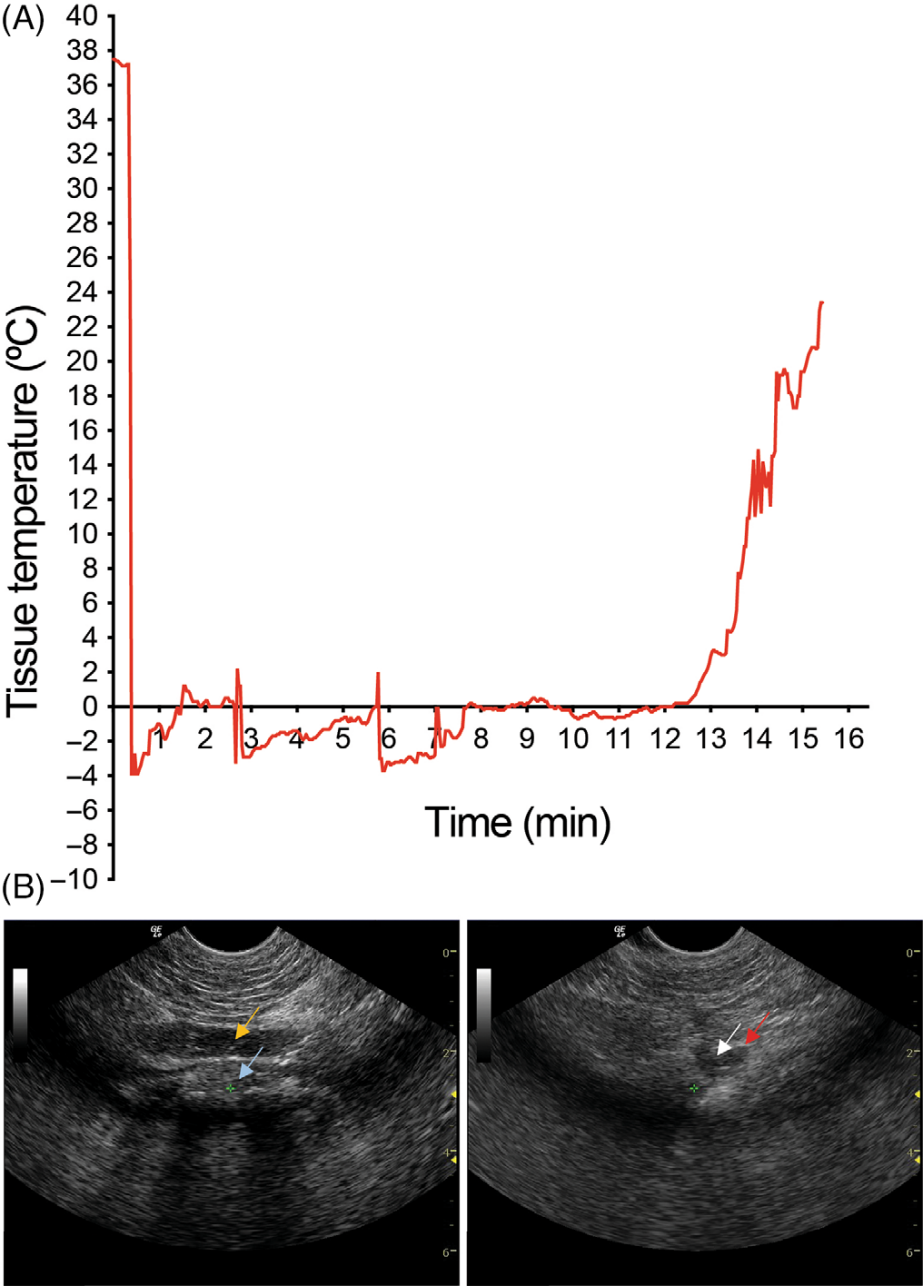
Ice ball formation with slurry injection at the base of the tongue was monitored with tissue temperature recording and ultrasound imaging. (A) Tissue temperature at the site of slurry injection at baseline and after injection. (B) Representative ultrasound images of the injection site in the base of the tongue before (left) and after (right) injection of slurry. Yellow arrow shows the hyoid bone, and blue arrow marks the base of the tongue. Red arrow shows the tip of the injection needle and white arrow marks the injected slurry at the base of the tongue

The animal study was approved by Massachusetts General Hospital IACUC and animals were housed at Massachusetts General Hospital Animal Facility in accordance with animal care regulations. Four Yorkshire female swine 4–6 months old were used in this study. Four Yorkshire swine in the test group were injected with ice‐slurry (−6°C). Slurry composed of normal saline (0.9% sodium chloride) and 10% glycerol was used as previously described.^12^ Ice‐slurry was made with a clinical‐grade ice‐slurry device developed in collaboration with a device engineering firm (Sage Product Development Inc., Foxborough, MA). Under general anesthesia and with ultrasound guidance, a 14 g needle was inserted through the anterior neck into the base of tongue. Anatomic landmarks on swine included the mandible and hyoid bone. The needle was inserted superior to the hyoid and guided into the left or right base of tongue by noting tissue displacement when the needle was passed perpendicular to the ultrasound probe. A laryngoscope with an appropriately sized Miller blade and rigid 0 degree laryngeal telescope were then used to confirm correct injection technique by visibly demonstrating the bulging of the tissue in the right or left base of tongue. A total of 60 ml ice‐slurry was injected during several minutes in three consecutive injections. This volume was selected as it has been determined by our group that this will consistently induce cooling of the tissue to a temperature of less than 10°C for at least 10 min (unpublished data). Tissue temperature at the site of injection was recorded using a thermocouple (Figure 1). The ice ball melted over time. The injected material dissipated over 10–15 min in all cases as noted on ultrasound and laryngoscopy. Extra fluid in all cases could be expressed from the needle with massage of the neck.

Animals were observed for 2 months for potential adverse effects, then sacrificed, and tongue tissue was harvested for gross and histologic analysis. This time frame was selected as prior cryolipolysis studies had demonstrated completion of the process within 2‐month time frame. Twelve biopsy specimens per swine were collected from the base of the tongue along a pre‐formed grid; these were fixed in 10% formalin, embedded in paraffin, sectioned at 5 μm, then stained with hematoxylin and eosin (H&E). Trichrome staining was used to examine collagen deposition.

Immunohistochemistry for neurofilaments and Luxol blue staining were performed to examine axons and myelin structure in nerves. A board‐certified pathologist assessed the biopsy sections by light microscopy for inflammatory and fibrotic changes in adipose tissue, muscles, nerves, vessels, salivary glands, lymphoid tissue and epithelium of the tongue.

## 3 RESULTS

Thermocouple recordings demonstrated that average local tissue temperature below 10°C for was achieved for 13.5 ± 1.1 minutes in all 4 swine with minimum tissue temperature of −4 ± 0.6 °C. After placement of the needle to the base of the tongue with ultrasound guidance, the injection took less than a minute to perform. In addition, the injection was very efficient in cooling the target tissue as it took seconds for the tissue temperature to reach subzero range. A representative thermocouple recording is shown in Figure 1A. Ultrasound imaging showed a hyperechoic ice‐ball formation at the injection site in the base of the tongue (Figure 1B), that diminished with ice melting. Successful injection into the base of tongue was confirmed with visualization of the ice and fluid filling the base of tongue on laryngoscopy. There were no complications upon injection, and no evidence of tongue necrosis or airway obstruction after injection.

**FIGURE 2:**
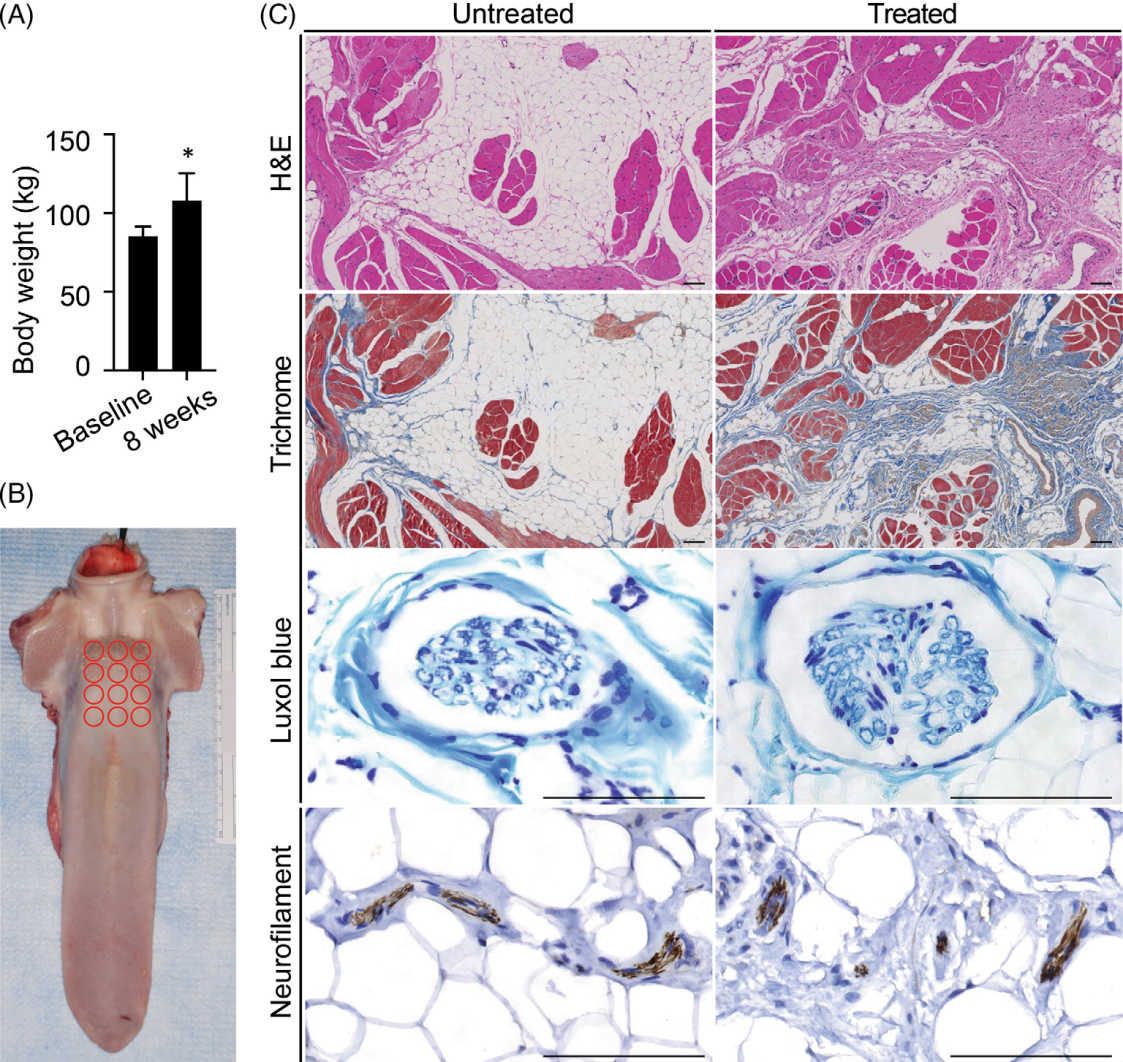
Injection of the slurry at the base of the tongue induced fat loss without surrounding tissue or nerve damage. (A) Graph shows body weight of animals at baseline and 8 weeks post injection. (B) Representative gross image of the tongue with biopsy sites at the base of the tongue marked in red. (C) Representative histological images of the base of the tongue at untreated sites, and sites injected with slurry. The H&E sections show adipose tissue admixed with muscle fibers. The treated panel shows adipose tissue reduction and increased collagen deposition (pink wavy areas in between darker muscle fibers within adipose tissue). Trichrome special stain decorates in royal blue normal collagen content in first 2 panels, and abundant collagen deposition in last panel on H&E section (treated panel). Luxol blue special stain highlights myelinated fibers, staining blue, in all panels without significant loss, and Neurofilament immunostain shows preserved axons, staining brown, in all three panels; scale bar, 100 μm. * represents the midline base of tongue. H&E, hematoxylin and eosin; RT, room temperature

Animals maintained normal food intake and weight gain post treatment, suggesting normal tongue function (Figure 2A). At 2 months post‐treatment, there was no evidence by gross tissue examination of scarring or trauma to the tongue (Figure 2B). In the twelve biopsy samples collected from each tongue at 2 months post treatment, there was histologic evidence of new collagen deposition in several of the sampled areas (Figure 2C). There were no histologic signs of damage to surrounding muscles, nerves or vessels. Immunohistochemistry and Luxol blue staining showed normal nerve structure, axons and myelin. No histologic signs of inflammation, scarring or necrosis were observed.

## 4 DISCUSSION

This is the first study demonstrating that ice‐slurry can be injected into the base of the tongue, causing the target tissue to rapidly reach temperatures capable of inducing cryolipolysis, leading to selective destruction of tongue fat without causing airway edema, obstruction or damage to the neurovascular structures of the tongue. Unlike topical cooling which can take over 45 minutes for fat tissue to reach below 10°C as heat needs to be extracted through layers of tissue, ice‐slurry injection delivered directly into fat allowed target tissue temperature to be reached within seconds. We successfully achieved temperatures below 10°C for greater than 10 min with a total dose of 60 ml utilizing a 10% glycerol slurry. Previous studies have shown that cooling induced cryolipolysis leads to loss of adipose tissue, increased collagen deposition and thickened fiberous septae within fat tissue.^8^, ^10^, ^12^ Adipose tissue loss and new collagen deposition was noted in many of the treated tongue tissue samples, which is consistent with previous histologic evidence of cryolipolysis.^8^

Some limitations of this feasibility study of a novel treatment include: use of an animal model, small sample size, and lack of quantifying fat loss, for example by imaging. We believe that the swine model is an appropriate one for humans based on the size, shape and fat content of the tongue. While pigs are obligate nasal breathers, significant base‐of‐tongue edema would still prevent adequate flipping of the epiglottis and would still potentially result in airway obstruction. Furthermore, swine have comparable neuroanatomy to the human tongue.^13^ In terms of limited assessment of efficacy, pigs grow at a significantly higher rate than adult humans. An adequately powered study demonstrating loss of fat in the pig model would require large numbers of swine which was beyond the scope of the pilot study. Instead, we chose to achieve a dose and cooling time shown in the literature to induce fat loss in multiple animal and human models to assess the feasibility of this treatment. Multiple prior studies show that cryolipolysis can be achieved by cooling the tissue to less than 10° C for about 10–15 min, but the correlation of fat volume loss with tissue temperature and duration is not known.^8^, ^10^, ^11^, ^12^

There are many treatments available for OSA. Liu et al.^3^ describe an update to the original Powell and Riley protocol. They recommend considering the patient phenotype followed by targeted therapy to the source of obstruction while considering the potentially high morbidity associated with many of the surgical procedures for sleep apnea. They also note that surgical therapy tends to fail in patients with high BMI. There is growing evidence that adipose tissue within the head and neck contributes to sleep apnea. According to Wang et al.,^5^ obese patients who underwent treatment to lose weight, reduction in base of tongue, retropharyngeal and parapharyngeal fat were strongly associated with reduction in AHI and improvement in sleep apnea, with tongue fat being the most significant factor. We selected the tongue as a specific subsite to start as we feel that its easily accessible and avoids potential involvement of the great vessels in the injection area. While injection near the carotid artery is not likely to injure the vessel, we believe the heat generated may limit our ability to cool the target tissue. It is well known that surgical treatments aside from tracheostomy in obese patients are often unsuccessful in the treatment of OSA. In addition, many obese patients with OSA have trouble losing weight due to lack of sleep, leading to less exercise.^14^ Ice‐slurry injection may represent a bridging therapy, allowing for ablation of fat in the tongue or potentially other areas of head and neck, allowing for reduction in OSA in this population. Although surgery for this population is not typically considered, with improved sleep, cognition and improved quality of life, these patients could lose further weight, which would improve their apnea and treatment options. Finally, cryolipolysis technology is likely to be far less painful than surgical treatments and is a quick minimally‐invasive injection which we believe could be ultimately used without general anesthesia. In the first‐in‐human study investigating the ice slurry injection for subcutaneous fat removal the mean pain score for ice slurry injected sites was 1.9/10 versus the 1.3/10 in control solution injected sites, suggesting that this will be a well‐tolerated procedure.^10^ The transcervical approach, previously used for radiofrequency ablation^15^ is ideal for this treatment as it allows for in office, injection of the slurry avoiding stimulation of the gag‐reflex and other disadvantages of an intra‐oral approach. This same approach has also been used with success in injection laryngoplasty. We believe that the injection could be performed via either ultrasound guidance or flexible fiberoptic laryngoscopy depending on the clinic resources. Decreased discomfort and post‐operative pain would potentially allow for multiple treatments in clinic if needed. Furthermore, injectable therapy would allow for rapid cooling of tissues reaching target temperature within seconds (Figure 1) when compared with topical cooling methods. Injectable therapy dissects along intramuscular tissue planes to allowing for select loss of target adipose tissue without damage to tongue. In this study, ice‐slurry injections were a minimally‐invasive, rapid, precise and efficient method of obtaining target adipose tissue cooling to the temperature range desired for inducing selective cryolipolysis.

## 5 CONCLUSION

Transcervical ultrasound‐guided ice‐slurry injection is feasible, safe and capable of selectively reducing adipose tissue in the base of the tongue, in a preclinical swine model without damage to the surrounding muscle, nerves or neurovascular components of the tongue or causing airway distress when properly injected. This method allows for selective removal of adipose tissue without damage to muscle, tongue mucosa or the complex neurovascular anatomy of the tongue, especially when compared to non‐selective techniques such as radiofrequency or cryoablation.^16^ Our findings suggest that injection of ice‐slurry in humans is a safe and potentially, minimally invasive treatment for OSA. We plan to conduct a first in human trial to determine safety and feasibility in humans.

## FUNDING INFORMATION

This work was supported by the Military Medicine Technology Transformation Collaborative, Henry M Jackson Foundation for the Advancement of Military Medicine, Inc. Award (HU0001‐17‐2‐0009). The Uniformed Services University of the Health Sciences (USU) is the awarding and administering office.

## CONFLICT OF INTEREST

R. Rox Anderson, Lilit Garibyan, and William Farinelli are inventors in patents related to this work, owned by the Massachusetts General Hospital. R. Rox Anderson, Lilit Garibyan, Sara Moradi Tuchayi, Ying Wang, and William Farinelli hold equity in a startup company which will commercialize this technology. The company had no involvement in this study. The authors declare no other competing interests.
